# Does the digital transformation of enterprises affect capital mismatch? evidence from Chinese listed firms

**DOI:** 10.1371/journal.pone.0313674

**Published:** 2025-02-03

**Authors:** Tianshan Yang, Kedong Wu, Xinming Du, Gonglin Yuan

**Affiliations:** 1 School of Finance and Economics, Nanning Vocational and Technical University, Nanning, China; 2 Faculty of Economics and Management, Luoyang Institute of Science and Technology, Luoyang, China; 3 School of Intelligent Manufacturing, Nanning Vocational and Technical University, Nanning, China; 4 School of Mathematics and Information Science, Center for Applied Mathematics of Guangxi, Guangxi University, Nanning, China; University of Almeria: Universidad de Almeria, SPAIN

## Abstract

In this study, based on the data of the Chinese listed firms, the effect of digital transformation on capital mismatch was examined. And the potential mechanism was also further discussed. It was found that digital transformation can significantly suppress capital mismatch, especially for non-state-owned enterprises, mature enterprises, and regions with high marketization and financial technology level. In addition, management capability and information environment are potential influencing mechanisms of digital transformation to suppress capital mismatch. These findings have important implications for revealing the relationship between enterprise digital transformation and capital mismatch, provides new ideas for improving the efficiency of capital allocation, and also provides important insights for enterprises to accelerate digital transformation and promote the high-quality development of enterprises.

## 1. Introduction

Digital transformation subverts the organizational structure, management concepts, and traditional governance techniques of traditional companies with big data, cloud computing, and artificial intelligence as the core technologies effectively improve the accuracy of public information. Thus, it can improve the corporate information environment [1], enhance internal and external corporate governance [2], boost the value and performance of enterprises [3], facilitating the increase of corporate total factor productivity. Numerous investigations and reports unveiled that enterprise digital transformation can significantly improve corporate social responsibility performance [4], reduce the concentration of customer resources [5], curb corporate expense stickiness and over-financialization [6,7], and increase corporate capacity utilization [8]. However, revolutionary changes in digital transformation can lead to some negative effects, such as increasing the complexity of business, affecting employee satisfaction, commitment, and organizational identification. These negative effects cause digital transformation to greater resistance, facing higher cost investment, and reducing the profit level of enterprises. Undoubtedly, there is still a great deal of uncertainty about the potential impact of digital transformation on enterprises. It is urgent to further explore in more depth in both academia and practice.

The efficiency of resource allocation is effective, and Pareto optimality can be achieved in a completely competitive market, while it fail in current actual market. In the context of increasing downward pressure on economy and society, specially, the phenomenon of resource misallocation is particularly prominent. Many countries are correcting the imbalance and misallocation of resource factors and improving the efficiency of resource allocation in the virtuous circle of national economy. It is well-known that capital mismatch is an important part of the field of resource mismatch and the most critical resource factor in the production activities of enterprises. Hsieh and Klenow [9] believed that the TFP of China’s manufacturing industry will increase by 30–50% if capital misallocation and labor misallocation can be eliminated. Bau and Matray [10] pointed out that the liberalization of foreign capital reduces the capital mismatch and improves the overall productivity of the industry. Fan and Chen [11] showed that the coverage, depth, and credit of digital finance can improve the efficiency of financial resource allocation. Li et al. [12] indicated that digital empowerment can significantly improve the efficiency of corporate resource allocation by reducing agency costs and improving operational capabilities. Hong et al. [13] found that digital transformation can significantly reduce the cost of equity capital of enterprises. Zhang and Wang [14] pointed out that digital economic development can significantly improve the level of capital mismatch. Besides, Han and Zhang [15] pointed out that internet development can directly improve the resource mismatch of regions with significant spatial spillover effects. There have been a large number of studies related to capital mismatch, however, it is found that there are fewer studies on the impact of digital transformation on a capital mismatch in the micro perspective of enterprises. Besides, it is not clear what impact effect exists between digital transformation and a capital mismatch at the micro-enterprise level. Therefore, this study aim at exploring the impact of digital transformation on capital allocation efficiency at the micro-firm level with a research sample of Chinese listed firms from 2010 to 2021. The results imply that digital transformation can significantly inhibit capital mismatch. Management capability and information environment are the potential influence mechanisms of enterprise digital transformation to inhibit capital mismatch. The inhibition of capital mismatch by enterprise digital transformation is more significant in non-state-owned enterprises, mature enterprises, and regions with high marketization and fintech levels.

The possible marginal contributions of this study to the existing literature are as follows: 1) To the best of our knowledge, the impact of digital transformation on capital mismatch is the first exploration in this study. Annual report data is used to describe the extent of digital transformation, and the impact of digital transformation on capital mismatch is empirically tested. The results suggest that digital transformation can significantly reduce corporate capital mismatch. 2) The impact of digital transformation on different types of enterprises is further explored. This study further distinguishes between state-owned enterprises, private enterprises, young enterprises, mature enterprises, enterprises in regions with high marketization, enterprises in regions with low marketization, enterprises in regions with strong fintech and enterprises in regions with weak fintech, and examines the role of digital transformation for different types of enterprises. The results reveal that digital transformation exhibit a greater impact on the capital mismatch of non-state-owned enterprises, mature enterprises, and enterprises in regions with high marketization degree and high fintech level. 3) Expanding the study on the channels of the impact of digital transformation on the capital mismatch of enterprises. The channels are explored through which digital transformation affects enterprise capital mismatch from the perspectives of management capability and information environment. The results unveil that digital transformation mainly reduces the capital mismatch of enterprises by improving both of management capabilities and improving the information environment.

## 2. Theoretical mechanisms and hypothesis development

Capital mismatch which is the capital is difficult to achieve the Pareto optimal state. This is due to capital cannot be based on the principle that marginal output equals marginal cost in the sector or enterprise free flow and allocation [16]. The capital mismatch is mainly derived from the information asymmetry between the supply and demand sides of capital, as well many market segmentation factors [14]. These could result in the emergence of adverse selection problems, increasing the cost of obtaining capital and investment, and making it difficult for capital factors to flow to sectors or enterprises with higher utilization efficiency. In the era of digital economy, digital technology has become an important underlying technology for scientific and technological progress in today’s world. The rapid development and wide application of digital technology profoundly affects the processing and circulation efficiency of information [17]. The digital transformation of enterprises breaks the space and time limitations, changes the market competition mode [18], enhances the ability of enterprises to tap the potential information value of a large amount of internal data. Therefore, digital transformation is a potential mode for the enterprise’s willingness to independently disclose information can be improved, which in turn transmits more effective information to the capital market to promote the formation of a high-quality digital network platform, thus effectively improving the enterprise information environment. On the one hand, the digital transformation of enterprises in the output of more effective information, the improvement of its own information output willingness will convey more available information to the capital market. In addition, the capital market entities outside the enterprise will inevitably grasp more adequate information than before, which can effectively judge the market credit demand and identify high-quality and high-efficiency enterprises through the digital technology [19]. Therefore, this can accurately invest capital in high-quality and high-efficiency enterprises, which reduces the financing of inefficient enterprises, inhibiting capital mismatch. On the other hand, enterprises can efficiently use digital technology to deal with massive internal and external data, and further encode and output them into standardized information. This can empower enterprise management, constrain the speculative behavior of the management, improve enterprise efficiency, and release the positive information about the high efficiency of the enterprise to the capital market through digital technology, so that the capital flows to the enterprise, thus inhibiting capital mismatch. Based on the above analysis, this paper proposes the following hypothesis:

**Hypothesis 1:** Digital transformation can significantly reduce capital mismatches.

Enterprise production and operation are largely affected by the ability of managers, who can decide the distribution and use of enterprise production factors, and the level of managerial ability directly affects whether the distribution of factors is scientific and reasonable and efficiency maximization. Enterprise digital transformation can empower enterprise management, improving enterprise managers for non-standardized, unstructured information integration and analysis capabilities. Thus, this makes enterprise managers more accurately determine the stage of development of the enterprise and the environment faced. This can guide production tasks with more quickly and accurately complete, improve the quality of products and services, and promote enterprise performance and productivity. Enterprise digital transformation can also build capital use monitoring platform and risk early warning platform for real-time dynamic monitoring and early warning of capital use, effectively constraining the management’s opportunistic behavior, improve the enterprise’s ability to manage the use of capital, and ultimately promote the improvement of enterprise efficiency. Additionally, the digital transformation of enterprises can also release positive signals of enterprise efficiency to the capital market while promoting enterprise efficiency, making high-efficiency enterprises more attractive and realizing the gradual flow of capital out of low-efficiency enterprises, and continuously gathering to high-efficiency enterprises, which can reduce the mismatch of enterprise capital. Based on this, this paper puts forward the following hypotheses:

**Hypothesis 2:** Digital transformation of enterprises effectively improves enterprise management capabilities, thus reducing capital mismatch.

The enterprise information environment is an important factor in the formation of capital mismatch, while digital transformation can alleviate information asymmetry and reduce market segmentation. On the one hand, there is an "information gap" between enterprises and external investors. The digital transformation can improve the integration capacity and quality of enterprise information to improve the information environment of enterprises, provide more high-quality information about enterprises for the capital supply side, reduce the information asymmetry and adverse selection between external capital supply and enterprises, which promote the free flow of capital. On the other hand, the process of enterprise digital transformation will also subconsciously affect the management thinking and internal control of enterprises. This improve the transparency of enterprise finance and management [20], improve the enterprise information environment, make it easier for capital to identify the real efficiency of enterprises, thus promoting the flow of capital out of the inefficient enterprises, and continuously gather to the high-efficiency enterprises. Therefore, it can reduce the capital mismatch in enterprises. Based on the above analysis, this paper proposes the following hypotheses:

**Hypothesis 3:** The digital transformation of enterprises effectively improves the information environment of enterprises, thus reducing capital mismatch.

## 3. Econometric model, variables and data description

### 3.1. Econometric model

The impact of digital transformation on corporate capital mismatch is further studied by following econometric model:

$$MisK_{it}=\alpha_{0}+\alpha_{1}Digital_{it}+\sum_{j}\beta_{j}X_{it}^{j}+\mu_{i}+\nu_{t}+\varepsilon_{it}$$ (1)

where the subscripts *i* and *t* are a particular firm and year, respectively; *MisK* is indicated corporate capital mismatch; *Digital* is indicated for digital transformation; *X*^*j*^ indicates the other control variables that affects firm’s capital mismatch; *α*_0_ is the constant terms; *α*_1_ and *β*_*j*_ are coefficients; *μ* and *ν* represent individual and time fixed effects, respectively; *ε* is a random error terms.

### 3.2. Variables description

#### 3.2.1. Dependent variable

Capital mismatch (*MisK*), a dependent variable, is stemmed from the degree of capital distortion, which refers to the related research of Hsieh and Klenow [9]. In this study, the firm’s operating income, net fixed assets, and labor compensation are utilized to estimate the firm’s output, capital input, and labor input, respectively [21].

#### 3.2.2. Independent variable

Digital transformation is the process, in which companies utilize digital technologies and hardware systems to increase efficiency and value in their production activities. Currently, digital transformation is widely used to estimate by corporate annual reports text analysis methods [22]. In this study, therefore, the Python technique is adopt to gather and organize the annual reports in Chinese listed companies, and the Java PDFbox library is used to extract all text contents. Besides, Based on the text content of the annual report, the word frequency is calculated from five dimensions, including artificial intelligence technology, blockchain technology, cloud computing technology, big data technology, and digital technology application. Furthermore, the natural logarithm of the total number of word frequencies plus one is used to calculate the digital transformation of enterprises. The specific digital transformation terms involved in the five dimensions are shown in Table 1.

**Table 1:** Digital transformation specific vocabulary.

Dimension	
Artificial Intelligence Technology	Speech Recognition, Face Recognition, Artificial Intelligence, Intelligent Robotics, Biometrics, Machine Learning, Business Intelligence, Natural Language Processing, Deep Learning, Autonomous Driving, Identity Verification, Image Understanding, Intelligent Data Analytics, Semantic Search
Blockchain Technology	Alliance Chain, Digital Currency, Decentralization, Smart Contracts, Distributed Computing, Bitcoin, Consensus Mechanisms, Differential Privacy Technology
Cloud Computing Technology	Internet of Things, Cloud Computing, Green Computing, Graph Computing, Streaming Computing, Converged Architecture, Cognitive Computing, Information Physical Systems, In-Memory Computing, Multi-Party Secure Computing, EB Scale Storage, Billion Level Concurrency, Brain-Like Computing
Big Data Technology	Big Data, Data Mining, Credit, Data Visualization, Virtual Reality, Augmented Reality, Mixed Reality, Text Mining, Heterogeneous Data
Digital Technology Application	E-commerce, Mobile Internet, Internet Finance, Mobile Internet, Mobile Payment, Fintech, Smart Customer Service, Digital Finance, Open Banking, Intelligent Investment Advisor, Intelligent Agriculture, Digital Marketing, Intelligent Marketing, B2B, Smart Home, Industrial Internet, Internet Connectivity, Smart Wearable, Intelligent Transportation, Internet Healthcare, B2C, Unmanned Retailing, Intelligent Grid, Third-Party Payment, O2O, Intelligent Environmental Protection, Intelligent Medical Care, Intelligent Energy, C2B, C2C, NFC Payment, Intelligent Culture and Travel, Quantitative Finance, Fintech

#### 3.2.3. Control variables

Capital mismatches may also be affected by other potential factors, and the following control variables are considered in this study: Firm size (Size), which is gauged by the natural logarithm of the firm’s total assets; Asset profitability (Roa), which is gauged by the ratio of net profit to total assets; Growth, expressed as the growth rate of operating income; Cash flow (Fcash), which is gauged by the ratio of net cash flow from operating activities; Board Independence (Indep), which is gauged by the ratio of the number of independent directors to the number of directors; Audit Quality (Big4), which is gauged by whether the auditors are from the Big 4 accounting firms; Gearing Ratio (Lev), which is gauged by the ratio of total liabilities to total assets; Shrhold, which is gauged by the sum of the shareholding ratios of the company’s top 3 major shareholders; Corporate Age, which is gauged by the natural logarithm of the difference of year minus establishment year plus one.

### 3.3. Data sources and description

In this study, all data are taken from China Stock Market & Accounting Research Database (CSMAR), which is related to Chinese listed companies from 2010 to 2021. Following the existing research practices [23,24], the sample data are processed as follows. a) The samples of ST, *ST and PT are removed; b) The samples with financial anomalies and missing data are deleted. c) The samples of the financial industry are excluded. In addition, winsorize was performed on all continuous variables at the 1% and 99% levels, minimizing the potential impact of results. The descriptive statistics of the main variables are shown in Table 2.

**Table 2:** Descriptive statistics.

Variable	Obs	Mean	Std. Dev.	Min	Max
MisK	35254	0.9836	0.4103	0.0492	2.2573
Digital	35254	1.3054	1.3768	0.0000	4.9767
Size	35254	22.1425	1.3243	19.0964	27.0802
Roa	35254	0.0378	0.0625	-0.2606	0.2003
Growth	35254	0.1813	0.4069	-0.5454	2.4952
Fcash	35254	0.0444	0.0710	-0.1872	0.2515
Indep	35254	0.3757	0.0534	0.3333	0.5714
Big4	35254	0.0574	0.2326	0.0000	1.0000
Lev	35254	0.4187	0.2090	0.0533	0.9226
Shrhold	35254	49.0603	15.5204	16.7164	87.2914
Age	35254	2.8732	0.3452	1.7918	3.5264

## 4. Regression results

### 4.1. Baseline regression analyses

Furthermore, The impact of enterprise digital transformation on capital mismatch is examined according to the Eq (1) with a fixed enterprise individual and time effects. while the method of gradually adding control variables is used for regression, and the regression results are shown in Table 3. As shown in columns (1)-(4) at Table 3, the enterprise digital transformation (Digital) exhibites significantly negative at 1% level, which suggests that Digital can significantly inhibit capital mismatch, verifying the reasonability of Hypothesis 1. As unveiled in Table 3, additionally, compared with column (1) without adding any control variable, the results of columns (2)-(4) with adding control variables did not fluctuate significantly, revealing a relatively small coefficient of Digital varies within the range of -0.0110 to -0.0081. This result also suggests that it is robust to significantly inhibit capital mismatch by enterprise digital transformation. In terms of economic significance, as displayed in column (4) at Table 3, capital mismatch will be reduced by 0.0112 (1.3768*0.0081) on average with increase of one standard deviation in corporate digital transformation (1.3768). Namly, it is equivalent to 2.73% of the standard deviation of capital mismatch in the sample (0.0112/0.4103). Therefore, the corporate digital transformation shows great significance of both Statistics and economics toward suppressing capital mismatch.

**Table 3:** Baseline regression results.

	(1)	(2)	(3)	(4)
	MisK	MisK	MisK	MisK
Digital	-0.0110***	-0.0085***	-0.0085***	-0.0081***
	(0.0029)	(0.0029)	(0.0029)	(0.0029)
Size		-0.0289***	-0.0292***	-0.0270***
		(0.0075)	(0.0075)	(0.0079)
Roa		0.4160***	0.4266***	0.3849***
		(0.0378)	(0.0382)	(0.0407)
Growth		0.0277***	0.0277***	0.0279***
		(0.0046)	(0.0046)	(0.0047)
Fcash			-0.0425	-0.0332
			(0.0264)	(0.0261)
Indep			-0.0356	-0.0294
			(0.0513)	(0.0508)
Big4			0.0007	0.0001
			(0.0165)	(0.0164)
Lev				-0.0268
				(0.0243)
Shrhold				0.0008*
				(0.0004)
Age				-0.0829**
				(0.0401)
_cons	1.0194***	1.6003***	1.6200***	1.7481***
	(0.0065)	(0.1607)	(0.1655)	(0.1978)
Year FE	Yes	Yes	Yes	Yes
Firm FE	Yes	Yes	Yes	Yes
N	35254	35254	35254	35254
R^2^	0.0108	0.0362	0.0364	0.0389

Note: Robust standard errors in parentheses

* *p* < 0.10

** *p* < 0.05

*** *p* < 0.01. The latter table is the same.

### 4.2. Discussion of endogeneity

In the baseline regressions, however, the potential endogeneity issue still cannot be fully overcame, although the characteristics, year and industry fixed effects of firm were controlled was controlled. Furthermore, inspired by related reports [25,26], the average digital transformation of other firms in the same industry and the digital transformation lag of one order are adopted as instrumental variables. Table 4 shows the results of instrumental variable (IV) estimation. The first-stage regression results are shown in columns (1) and (3) of Table 4. The coefficients of the average digital transformation of other firms in the same industry and the digital transformation lag of one order are statistically significant and positive, at the 1% level. This indicate that the average digital transformation of other firms in the same industry and the digital transformation lag of one order are positively correlated with digital transformation. Additionally, the values of the Cragg-Donald Wald F-statistics are greater than the critical value of 16.38, indicating that there is no weak IV selection problem in this study. The Kleibergen-Paap rk LM test rejected the instrumental variable unidentifiable hypothesis at the 1% level, indicating the selected instrumental variables are identifiable. The second-stage regression results are shown in columns (2) and (4) of Table 4. The coefficients of digital transformation are statistically significant and negative, at the 1% level. This indicates that the main conclusions still are tenable after considering the endogeneity issue.

**Table 4:** Robustness testing using instrumental variable method.

	(1)	(2)	(3)	(4)
	Digital	MisK	Digital	MisK
Digital		-0.7390***		-0.0263***
		(0.0784)		(0.0065)
Digital_IndOther	0.3985***			
	(0.0323)			
Digital_IV			0.4716***	
			(0.0081)	
Size	0.2689***	0.1795***	0.1590***	-0.0224***
	(0.0200)	(0.0270)	(0.0142)	(0.0082)
Roa	-0.0728	0.2702***	0.1332	0.3581***
	(0.1136)	(0.0897)	(0.0883)	(0.0429)
Growth	0.0254**	0.0502***	0.0514***	0.0323***
	(0.0119)	(0.0100)	(0.0111)	(0.0049)
Fcash	-0.1136	-0.1117*	-0.0816	-0.0075
	(0.0809)	(0.0653)	(0.0703)	(0.0276)
Indep	-0.5135***	-0.4228***	-0.4096***	-0.0341
	(0.1649)	(0.1355)	(0.1225)	(0.0491)
Big4	-0.0214	-0.0064	-0.0092	-0.0046
	(0.0568)	(0.0423)	(0.0408)	(0.0164)
Lev	-0.1006	-0.1197**	-0.1092**	-0.0171
	(0.0748)	(0.0594)	(0.0530)	(0.0252)
Shrhold	-0.0062***	-0.0043***	-0.0022***	0.0004
	(0.0012)	(0.0011)	(0.0008)	(0.0004)
Age	-0.1029	-0.1810	-0.1714*	-0.0344
	(0.1417)	(0.1114)	(0.0891)	(0.0407)
_cons	-4.6901***	-1.6715***	-2.3633***	1.5598***
	(0.5258)	(0.5728)	(0.3668)	(0.2081)
Year FE	Yes	Yes	Yes	Yes
Firm FE	Yes	Yes	Yes	Yes
N	35245	35245	30246	30246
R^2^	0.3622		0.4722	
Under-identification test	292.960 [P-val<0.0001]		2866.761 [P-val<0.0001]	
Weak-identification test	603.936 <16.38>		7597.792 <16.38>	

### 4.3. Other robustness tests

1. Replacing explanatory variables. First, digital transformation is measured by replacing the natural logarithm of the sum of word frequencies in the annual report, respectively, denoted as Digital_a1. Second, replacing the digital transformation as the natural logarithm of the sum of the keywords of MD&A The natural logarithm of the sum is measured, denoted as Digital_a2. This is because of the information in the management’s discussion and analysis (MD&A) section highly concentrates on the operation of the enterprise, and the disclosure of the information is more serious and timely. While there is a certain degree of breadth in the information of the entire content of the annual report [27]. The regression results after replacing the digital transformation measure, as shown in columns (1) and (2) at Table 5, suggest that the coefficients of Digital_a1 and Digital_a2 are significantly negative at the 1% level, which demonstrates the robustness of the main findings of the main study after replacing the firm’s digital transformation measure.

**Table 5:** Replacing core variables and digital transformation breakdown.

	(1)	(2)	(3)	(4)	(5)	(6)	(7)
	MisK	MisK	MisK	MisK	MisK	MisK	MisK
Digital_a1	-0.0080***						
	(0.0028)						
Digital_a2		-0.0005***					
		(0.0001)					
lnAIT			-0.0150***				
			(0.0045)				
lnBlo				-0.0849***			
				(0.0236)			
lnClo					-0.0135***		
					(0.0044)		
lnBig						-0.0153***	
						(0.0041)	
lnDig							-0.0048
							(0.0032)
Size	-0.0347***	-0.0262***	-0.0270***	-0.0286***	-0.0271***	-0.0264***	-0.0283***
	(0.0085)	(0.0079)	(0.0079)	(0.0079)	(0.0079)	(0.0079)	(0.0079)
Roa	0.3847***	0.3886***	0.3821***	0.3824***	0.3846***	0.3824***	0.3856***
	(0.0500)	(0.0410)	(0.0407)	(0.0407)	(0.0407)	(0.0407)	(0.0407)
Growth	0.0316***	0.0277***	0.0273***	0.0275***	0.0276***	0.0277***	0.0279***
	(0.0058)	(0.0047)	(0.0047)	(0.0047)	(0.0047)	(0.0047)	(0.0047)
Fcash	-0.0388	-0.0310	-0.0333	-0.0339	-0.0336	-0.0340	-0.0327
	(0.0351)	(0.0262)	(0.0262)	(0.0261)	(0.0261)	(0.0262)	(0.0262)
Indep	-0.0492	-0.0317	-0.0288	-0.0253	-0.0291	-0.0281	-0.0268
	(0.0610)	(0.0513)	(0.0508)	(0.0506)	(0.0508)	(0.0506)	(0.0507)
Big4	-0.0046	0.0031	0.0008	0.0007	0.0004	0.0003	0.0001
	(0.0199)	(0.0163)	(0.0163)	(0.0164)	(0.0164)	(0.0163)	(0.0165)
Lev	0.0190	-0.0273	-0.0262	-0.0269	-0.0266	-0.0261	-0.0260
	(0.0301)	(0.0244)	(0.0243)	(0.0243)	(0.0243)	(0.0242)	(0.0244)
Shrhold	0.0002	0.0007	0.0007*	0.0008**	0.0008*	0.0007*	0.0008**
	(0.0005)	(0.0004)	(0.0004)	(0.0004)	(0.0004)	(0.0004)	(0.0004)
Age	-0.0029	-0.0839**	-0.0786**	-0.0796**	-0.0809**	-0.0803**	-0.0825**
	(0.0500)	(0.0403)	(0.0400)	(0.0401)	(0.0400)	(0.0400)	(0.0402)
_cons	1.7176***	1.7401***	1.7369***	1.7695***	1.7460***	1.7275***	1.7716***
	(0.2268)	(0.1989)	(0.1978)	(0.1976)	(0.1982)	(0.1971)	(0.1984)
Year FE	Yes	Yes	Yes	Yes	Yes	Yes	Yes
Firm FE	Yes	Yes	Yes	Yes	Yes	Yes	Yes
N	21755	34792	35254	35254	35254	35254	35254
R^2^	0.0394	0.0402	0.0394	0.0394	0.0393	0.0399	0.0382

2. Digital transformation decomposition. Thanks for digital transformation is involved to various technologies, digital transformation is classified into five categories including industrial intelligence technology, blockchain technology, cloud computing technology, big data technology, and digital technology application, to examine whether the main findings are robust or not. The regression results of the decomposed sub-dimensions are shown in columns (3) to (7) at Table 5 report. Except for the sub-dimension of digital technology applications, the regression results exhibit that all sub-dimension coefficients are negative and all the coefficients of all the dimensions are significant at the 1% level, suggesting that the main conclusions are tenable.

3. Extend the time observation window. It is well-known that digital transformation is significantly important toward the high-quality development of enterprises. In this study, to extend the time window in which digital transformation affects capital mismatch to test the robustness of the conclusions, digital transformation and capital mismatch do lag 1~3 periods and front 1~3 periods of treatment, respectively. It was found that, as shown in Table 6, the digital transformation coefficients are significantly negative at the 1% level for both the capital mismatch front-loading treatment and the digital transformation lagging treatment. These results further support the main conclusions as mentioned above.

**Table 6:** Extended time observation window.

	(1)	(2)	(3)	(4)	(5)	(6)
	F1MisK	F2MisK	F3MisK	MisK	MisK	MisK
Digital	-0.0132***	-0.0157***	-0.0140***			
	(0.0031)	(0.0033)	(0.0032)			
L1Digital				-0.0124***		
				(0.0031)		
L2Digital					-0.0149***	
					(0.0033)	
L3Digital						-0.0129***
						(0.0032)
Size	-0.0392***	-0.0424***	-0.0371***	-0.0266***	-0.0285***	-0.0294***
	(0.0084)	(0.0073)	(0.0066)	(0.0082)	(0.0086)	(0.0094)
Roa	0.1394***	0.0053	-0.0356	0.3546***	0.3386***	0.3385***
	(0.0453)	(0.0465)	(0.0459)	(0.0431)	(0.0432)	(0.0450)
Growth	0.0215***	0.0084**	0.0070	0.0310***	0.0321***	0.0278***
	(0.0039)	(0.0039)	(0.0043)	(0.0049)	(0.0051)	(0.0057)
Fcash	-0.0312	-0.0242	0.0098	-0.0054	0.0018	-0.0073
	(0.0259)	(0.0240)	(0.0259)	(0.0276)	(0.0273)	(0.0288)
Indep	-0.0093	0.0214	-0.0018	-0.0233	-0.0533	-0.0430
	(0.0509)	(0.0468)	(0.0442)	(0.0486)	(0.0473)	(0.0489)
Big4	0.0053	0.0076	0.0099	-0.0044	-0.0008	-0.0046
	(0.0179)	(0.0175)	(0.0147)	(0.0166)	(0.0173)	(0.0179)
Lev	0.0096	0.0079	0.0297	-0.0143	-0.0148	-0.0008
	(0.0264)	(0.0248)	(0.0238)	(0.0253)	(0.0266)	(0.0284)
Shrhold	0.0003	0.0002	0.0003	0.0005	0.0002	0.0003
	(0.0004)	(0.0004)	(0.0004)	(0.0004)	(0.0004)	(0.0005)
Age	-0.0359	0.0088	0.0052	-0.0299	0.0209	0.0261
	(0.0399)	(0.0408)	(0.0430)	(0.0407)	(0.0426)	(0.0475)
_cons	1.9047***	1.8579***	1.7386***	1.6220***	1.5547***	1.5394***
	(0.2060)	(0.1830)	(0.1712)	(0.2075)	(0.2203)	(0.2438)
Year FE	Yes	Yes	Yes	Yes	Yes	Yes
Firm FE	Yes	Yes	Yes	Yes	Yes	Yes
N	30246	26185	22576	30246	26185	22576
R^2^	0.0235	0.0225	0.0159	0.0372	0.0403	0.0350

### 4.4. Heterogeneity analysis

The heterogeneous toward the impact of firms’ digital transformation in inhibiting capital mismatch on the nature of ownership, growth period, degree of marketization, and FinTech development were further explored. In this study, the samples were firstly divided into state-owned enterprises (SOEs) and non-state-owned enterprises (NSOEs) for estimation based on the nature of the actual controller of the firms. The results, as exhibited in columns (1) and (2) at Table 7, reveal that the coefficient of digital transformation is negative for both SOEs and non-SOEs. The coefficient of -0.0050 for the SOE group fails the statistical significance test, while the coefficient of -0.0084 for the non-SOE group shows significant at the 5% level. This may be due to the state-owned enterprises in the market competition has the advantage of national credibility, ensuring that many resources have the advantage of access to the market to reduce the difficulty of development. Thus, there is less competitive pressure to the state-owned enterprises in the market. This leads to less innovation and transformation of the development of the power compared with the non-state-owned enterprises, resulting in dully capturing the huge potential benefits of cutting-edge digital technology, and less willingness to promote the digital transformation of the. However, non-state-owned enterprises do not have national credibility embedded in the market competition, facing the huge pressure of not advancing or retreating. To increase the share of enterprises in the market, therefore, non-state-owned enterprises exhibit a keen insight. They recognize that the digitalization of the enterprise can be empowered by the development of the technological innovation and transformation of the activities with more stronger subjective initiative. Therefore, compared to SOEs, non-SOEs have stronger motivation and higher willingness to promote digital transformation on the ground. They try to improve the operational efficiency and value of the enterprise through digital transformation, releasing more positive signals to the outside world of the high efficiency of the enterprise to alleviate the capital mismatch. What follows, the samples were divided into two groups of young and mature firms based on whether the age of the firm is greater than the median. Namely, firms with age greater than the median are mature firms (assigned a value of 1), and firms with age less than the median are young firms (assigned a value of 0). As displayed in columns (3) and (4) at Table 7, it is found that the coefficient of digital transformation is negative for both young and mature firms, while only in the mature firms group does the coefficient of digital transformation pass the statistical significance test. This may profit from the reserve of management ability and technical ability of strong talent during the long-term management in mature enterprises, which can keenly identify and implement the digital transformation. Besides, it is more able to use the digital transformation empowered enterprise capital allocation, improving the efficiency of capital allocation, and reducing capital mismatch. Again, the samples were divided into two groups of high and low marketability regions with the Fanzang marketability index, based on whether the marketability of the enterprise location is greater than the median. Specially, the marketability is greater than the median for the high marketability region group, otherwise for the low marketability region group. The regression results are shown in columns (5) and (6) at Table 7, implying that the coefficient of digital transformation of firms in the high marketability region group is significantly negative at the 5% level. While the coefficient in the low marketability group is negative but fails the significance test, suggesting that there is a difference in the inhibition of capital mismatch by digital transformation of firms in terms of the degree of marketability of firms’ locations. This suggests that, in the process of capital allocation, digital transformation as a technological complement to market regulation is more likely to form a technological complementary force in regions with high marketization degree, empowering capital allocation and reducing the degree of capital mismatch. Finally, the samples were divided into two groups with Peking University’s Digital Inclusion Index. Regarding the digital inclusion index above the median for the location of the enterprise as a strong fintech regional group, and otherwise as a weak fintech regional group. The group regression results are displayed in columns (7) and (8) at Table 7, which reveal that the coefficient of firms’ digital transformation in strong fintech regions is significantly negative at the 1% level. While the coefficient of the group of weak fintech regions is negative but not significant. This may be stemmed from the firms’ digital transformation needs financial support, and the high level of development of fintech in the region has facilitated the financing of the firms and pushed them to carry out the digital transformation, which is conducive to unleashing the strength of the firms’ digital transformation in suppressing the financial mismatch.

**Table 7:** Heterogeneity analysis.

	(1)	(2)	(3)	(4)	(5)	(6)	(7)	(8)
	SOEs	NSOEs	Young	Mature	High marketability	Low marketability	strong fintech	weak fintech
Digital	-0.0050	-0.0084**	-0.0046	-0.0086*	-0.0092**	-0.0043	-0.0115***	-0.0037
	(0.0048)	(0.0036)	(0.0035)	(0.0045)	(0.0038)	(0.0041)	(0.0038)	(0.0043)
Size	-0.0148	-0.0319***	-0.0285***	-0.0305**	-0.0365***	-0.0181*	-0.0263**	-0.0247*
	(0.0156)	(0.0096)	(0.0095)	(0.0121)	(0.0114)	(0.0109)	(0.0110)	(0.0127)
Roa	0.3780***	0.3929***	0.4120***	0.3218***	0.3157***	0.4390***	0.2976***	0.4421***
	(0.0737)	(0.0492)	(0.0515)	(0.0630)	(0.0604)	(0.0569)	(0.0696)	(0.0588)
Growth	0.0289***	0.0300***	0.0298***	0.0273***	0.0327***	0.0255***	0.0200***	0.0304***
	(0.0073)	(0.0061)	(0.0055)	(0.0069)	(0.0065)	(0.0062)	(0.0074)	(0.0070)
Fcash	-0.0258	-0.0194	-0.0208	-0.0467	-0.0171	-0.0452	0.0236	-0.0740*
	(0.0375)	(0.0351)	(0.0362)	(0.0362)	(0.0352)	(0.0364)	(0.0391)	(0.0392)
Indep	0.0427	-0.0726	-0.1378**	0.0985	-0.0138	-0.0398	-0.0434	0.0275
	(0.0693)	(0.0737)	(0.0663)	(0.0785)	(0.0844)	(0.0626)	(0.0660)	(0.0745)
Big4	0.0054	-0.0037	-0.0035	0.0074	0.0087	0.0117	-0.0100	0.0144
	(0.0214)	(0.0235)	(0.0199)	(0.0261)	(0.0191)	(0.0194)	(0.0208)	(0.0296)
Lev	0.0078	-0.0433	-0.0283	-0.0352	-0.0190	-0.0322	-0.0357	-0.0183
	(0.0444)	(0.0294)	(0.0296)	(0.0380)	(0.0312)	(0.0356)	(0.0339)	(0.0403)
Shrhold	-0.0001	0.0009*	0.0014**	0.0002	0.0009*	0.0002	0.0005	0.0006
	(0.0006)	(0.0005)	(0.0005)	(0.0006)	(0.0005)	(0.0007)	(0.0005)	(0.0007)
Age	-0.0066	-0.1015*	-0.0526	-0.4293**	-0.0239	-0.1536**	0.0272	-0.1758**
	(0.0495)	(0.0567)	(0.0552)	(0.1699)	(0.0454)	(0.0673)	(0.0476)	(0.0703)
_cons	1.2810***	1.9137***	1.7106***	2.8028***	1.7987***	1.7662***	1.4730***	1.9685***
	(0.3817)	(0.2483)	(0.2392)	(0.5778)	(0.2584)	(0.2959)	(0.2645)	(0.3327)
Year FE	Yes	Yes	Yes	Yes	Yes	Yes	Yes	Yes
Firm FE	Yes	Yes	Yes	Yes	Yes	Yes	Yes	Yes
N	12029	22571	19070	16184	17034	18212	15048	14089
R^2^	0.0225	0.0499	0.0603	0.0280	0.0490	0.0359	0.0367	0.0416

## 5. Influence channel identification test

There are numerous reports discussed the impact of corporate digital transformation on capital mismatch, while it needs to further explore toward what aspects of corporate digital transformation affect capital mismatch. For this, both of the management capability effect and the information improvement effect are further examined in this study. If corporate digital transformation reduces corporate capital mismatches through the management capability effect and the information improvement effect, then the effect of corporate digital transformation in reducing capital mismatches will be more pronounced in firms with lower management capabilities and poorer information environments [28]. Therefore, the econometric model (2) is designed to test the mechanism of corporate digital transformation in curbing capital mismatch:

$$MisK_{it}=\alpha_{0}+\alpha_{1}Digital_{it}+\alpha_{2}Digital_{it}\times MIDum_{it}+\alpha_{3}MIDum_{it}+\sum_{j}\beta_{j}X_{it}^{j}+\mu_{i}+\nu_{t}+\varepsilon_{it}$$ (2)

where *MIDum* is substituted for managerial ability and information environment variables; *α*_2_ and *α*_3_ are coefficients, and other variables are set the same as in (1).

In terms of management capability effect, the inventory turnover (AC) is uesed as a proxy variable for firms’ management capability. This is due to the inventory turnover ratio is equal to the cost of goods sold divided by the average inventory, indicating the average number of times an enterprise turns over its inventory. A higher value means a faster inventory turnover, a lower level of inventory occupancy, a higher level of operational efficiency, a higher level of capital management efficiency, and therefore a higher level of managerial competence of the enterprise [29]. In this paper, the inventory turnover ratio less than the median are regarded as a weak management capability enterprise, i.e., AC is 1, otherwise it is regarded as a strong management capability enterprise, and AC is assigned a value of 0. As shown in column (1) at Table 8, the regression results unveil that the coefficient of the cross-multiplier term of enterprise’s digital transformation and inventory turnover is negative and significant at 1% level. This suggests that, relative to enterprises with strong management capabilities, enterprise digital transformation is more conducive to promoting the reduction of capital mismatches in enterprises with weaker management capabilities, when keeps other conditions unchanged. For instance, enterprise digital transformation exerts the management capability effect, which verifies the conclusion of Hypothesis 2.

**Table 8:** Mechanism test analysis.

	(1)	(2)
	MisK	MisK
Digital	-0.0030	-0.0051
	(0.0033)	(0.0031)
Digital_ACDum	-0.0106***	
	(0.0033)	
Digital_InfoDum		-0.0059**
		(0.0024)
ACDum	-0.0039	
	(0.0068)	
InfoDum		0.0046
		(0.0042)
Size	-0.0265***	-0.0266***
	(0.0079)	(0.0079)
Roa	0.3833***	0.3871***
	(0.0406)	(0.0406)
Growth	0.0272***	0.0279***
	(0.0047)	(0.0047)
Fcash	-0.0397	-0.0337
	(0.0266)	(0.0261)
Indep	-0.0287	-0.0311
	(0.0509)	(0.0507)
Big4	-0.0005	0.0006
	(0.0164)	(0.0163)
Lev	-0.0254	-0.0274
	(0.0243)	(0.0242)
Shrhold	0.0008*	0.0008*
	(0.0004)	(0.0004)
Age	-0.0807**	-0.0825**
	(0.0400)	(0.0401)
_cons	1.7347***	1.7366***
	(0.1976)	(0.1978)
Year FE	Yes	Yes
Firm FE	Yes	Yes
N	35254	35254
R^2^	0.0413	0.0394

In terms of information improvement effect, this paper draws on existing studies [30] to use stock turnover rate (Info) as a proxy variable for information environment, with higher stock turnover rate indicating better information environment. In this paper, the stock turnover rate less than the median is regarded as poor information environment firms (Info is assigned a value of 1), otherwise it is regarded as good information environment firms (Info is assigned a value of 0). The regression results are shown in column (2) at Table 8, which suggest that, at the 5% level, the coefficients of the cross-multiplier terms of enterprise digital transformation and stock turnover are all negative and significant. This indicates that, relative to firms with a good information environment, enterprise digital transformation is more conducive to promoting firms with a poor information environment to reduce capital mismatch, when keeps other conditions unchanged. For instance, enterprise digital transformation exerts the information environment improvement effect, which verifies the conclusion of Hypothesis 3.

## 6. Conclusions and implications

### 6.1 Main findings

Within the context of the rapid development of digital transformation, the main strategic direction of China’s economic and social development is the integration of digitalization with the real economy. Digital transformation helps to strengthen corporate governance, stimulate sustainable and healthy corporate development, increase the value of companies, and improve the capital market information environment. This paper empirically investigates the impact of enterprise digital transformation on capital mismatch and its mechanism using a sample of Chinese A-share listed companies from 2010 to 2021. This study evidences some conclusions in several aspects: i) Enterprise digital transformation can significantly inhibit capital mismatch. It is found that the impact of digital transformation has a more integrated and comprehensive character, which is different from the impact of restraining capital misallocation in the development of the Internet [31], digital finance [32], etc. ii) Management capability and information environment are potential factors for enterprise digital transformation to inhibit capital mismatch. Compared with the factors affecting the quality of internal control [33], the digital transformation is more inclined to restrain the opportunistic behavior of management, improve the management ability of enterprises to use capital, and release positive signals of enterprise efficiency to the capital market iii) Enterprise digital transformation to inhibit capital mismatch is more significant in non-state-owned enterprises, mature enterprises, and regions with high marketization and fintech level. Most reported studies focus more on the heterogeneity of enterprise, such as the nature and maturity of enterprise property rights and the degree of marketization [34], while the performance of regional fintech level development has expanded in this study.

### 6.2 Managerial implications

On the one hand, the behavior with respect to the functions of the government and related administrative departments need to be actively carried out. First, they should actively carry out digital change, accurately grasp the new opportunities for the development of digital transformation, improve the system of laws and regulations in the field of digitalization, increase efforts to protect the intellectual property rights of digital technology, and stimulate the potential and vitality of the digital development of enterprises. Besides, it is important to improve the policy support system, and introduce targeted and special financial and tax policies and subsidies to promote enterprises to accelerate the completion of intelligent transformation and it will also actively build big data sharing platforms and big data centers, improve digital infrastructure, and promote the seamless connection between enterprise resources and internet platforms. It will also strive to improve the business environment for digital applications, improve the digital intellectual property rights trading market, and create conditions for the trading and application of enterprises’ digital technologies. At the same time, they should also pay attention to the "precise drip irrigation" of enterprises’ digital transformation, and take into full consideration the need for the "precise drip irrigation" of enterprises. In addition, it is also necessary to focus on the "precise drip irrigation" of enterprise digital transformation, give full consideration to digital transformation to curb the heterogeneous effect of capital mismatch in non-state-owned enterprises, mature enterprises, the degree of marketization and the high level of financial technology in the region. Moreover, in order to give full play to the spillover effect of its successful digital transformation experience, it is also significant to support the development of targeted batches of industry-leading enterprises with a good foundation and strong strength to explore the digitization of the intelligent operation of the new model.

On the other hand, the keen initiative of the enterprises should be actively exerted. enterprises should pay attention to the development of digital transformation, take the initiative to respond to digital development changes, actively carry out digital innovation and the introduction of digital technology, increase the training of enterprise management in the application of digital technology to improve the management’s digital management capabilities. Besides, it is also important to increase the construction of the enterprise’s internal data integration platform and the training of relevant personnel to improve the production and operation of the enterprise to enhance the sharing of information and enhance the enterprise’s various departments of production and operation of the synergistic effect, and improve the production and operation of the enterprise. Additionally, the synergistic effect is watchable to improve the production efficiency of the enterprise, and create enterprise visibility. Moreover, to inhibit the mismatch of capital, it is essential to accelerate the establishment of a sound information disclosure system, strengthen the standardization and structuring of enterprise information disclosure, reduce the information cost of investors in identifying the enterprise, improve the efficiency of financial institutions in identifying the enterprise, and maximize the release of the positive effect of the enterprise’s management ability and information environment on digital transformation.

### 6.3 Limitations and future research

There are also some limitations to this study, which need to be conducted in the future. For instance, digital transformation is a gradual process, and digital resources become important strategic resources for companies must have non-replicable and unique digital capabilities. Empirical analysis cannot recognize the process of transitioning digital resources to digital capabilities, which can be tested in the future using case studies or questionnaires. Additionally, this study discusses the impact of digital transformation on capital mismatch, but the impact of digital transformation on high-quality development of capital and labor markets still needs to be included in the whole framework in future research. Moreover, this study only focuses on the impact mechanisms of both managerial capability and information environment. Therefore, in future study, the factors such as government policies, competitive strategies, and external supervision should take into consideration to deepen our understanding of the impact of digital transformation on capital mismatch.

## Supporting information

S1 Data (XLS)
